# Rachitis

**Published:** 1841-01

**Authors:** 


### RACHITIS.

No apology, we are sure, need be offered to our readers for the great length of Dr. Colescott's translation of the celebrated memoir of M. Guérin, which occupies so large a portion of the present number of the Journal. It is one of a series of articles on the deformities of the osseous system, comprised in a beautiful octavo volume of upwards of four hundred closely printed pages, with here and there an illustrative lithographic engraving, which was published at Paris in 1839. One of these papers—that on the general etiology of congenital club-foot—appeared, not long ago, in Professor Dunglison's "Medical Library;" and, if we mistake not, that which is now presented to the American profession will be equally acceptable to them. Indeed, it may justly be regarded as the most valuable of them all. Much, it is true, had been previously written upon the subject, but we perfectly agree with the learned author when he declares that no one had thoroughly understood it. This, in fact, appears at once obvious from the great variety of diseases which have been described, from time to time, under the name of rachitis, which seems to have hitherto been employed rather in a generic than a specific sense, precisely as the term "phthisis" was lately used to designate a whole group of dissimilar affections, and not, as is now done, a single malady, marked by peculiar symptoms and distinct anatomical characters. It was reserved for M. Guérin, the distinguished director of the orthopedic Institute of Paris, to place the disease in its true light, by studying it as it occurs in the different periods of life, in both sexes, in different orders of society, upon the living subject, in the dead body, and in the different stages of its progress; thus dissipating, by a series of the most admirably conducted observations and dissections, the confusion which had heretofore existed in relation to the subject, at the same time that he has presented us with a large amount of the most valuable information, which may always be referred to with interest and instruction.

In our next number we shall endeavor to present more variety, which is, perhaps, not less the spice of medical literature and sci-science than of life. Within the last few months, several interesting articles have been received, intended for the present number, which have been necessarily laid over for want of room. They will appear as early as practicable. The review of Professor Gross' Elements, which has been for sometime suspended, will also be resumed in the next, or an early number. D.

